# Carbohydrate mouth rinsing has no effect on power output during cycling in a glycogen-reduced state

**DOI:** 10.1186/s12970-016-0131-1

**Published:** 2016-04-23

**Authors:** Ajmol Ali, Michelle Ji Yeon Yoo, Catherine Moss, Bernhard H. Breier

**Affiliations:** School of Sport and Exercise, Massey University, Auckland, New Zealand; School of Applied Sciences, Auckland University of Technology, Auckland, New Zealand; School of Food and Nutrition, Massey University, Auckland, New Zealand

**Keywords:** Ergogenic aid, Fluid ingestion, Time trial, Sports drink, Mouthwash, Supplementation

## Abstract

**Background:**

The effect of mouth rinsing with a carbohydrate (CHO) solution on exercise performance is inconclusive with no benefits observed in the fed state. This study examined the effect of CHO mouth rinse or CHO ingestion on performance in 9 moderately trained male cyclists.

**Methods:**

Four trials were undertaken, separated by 7 days, in a randomized, counterbalanced design. Each trial included a 90-min glycogen-reducing exercise protocol, immediately followed by a low CHO meal and subsequent overnight fast; the following morning a 1-h cycling time trial was conducted. The trials included 15 % CHO mouth rinse (CHOR), 7.5 % CHO ingestion (CHOI), placebo mouth rinse and placebo ingestion. Solutions were provided after every 12.5 % of completed exercise: 1.5 mL · kg^−1^ and 0.33 mL · kg^−1^ body mass during ingestion and rinse trials, respectively. During rinse trials participants swirled the solution for 8 s before expectorating. Blood samples were taken at regular intervals before and during exercise.

**Results:**

Performance time was not different between trials (*P* = 0.21) but the 4.5-5.2 % difference between CHOI and other trials showed moderate practical significance (Cohen’s d 0.57-0.65). Power output was higher in CHOI relative to other trials (*P* < 0.01). There were no differences between CHOR and placebo groups for any performance variables. Plasma glucose, insulin and lactate concentrations were higher in CHOI relative to other groups (*P* < 0.05).

**Conclusions:**

In a fasted and glycogen-reduced state ingestion of a CHO solution during high-intensity exercise enhanced performance through stimulation of insulin-mediated glucose uptake. The CHO mouth rinsing had neither ergogenic effects nor changes in endocrine or metabolic responses relative to placebo.

## Background

The ergogenic effects of carbohydrate (CHO) ingestion during prolonged exercise are well known. With CHO ingestion, better maintenance of blood glucose [1], increase in CHO oxidation [1] and/or sparing of muscle glycogen during intermittent [2] and continuous [3] high-intensity exercise have been shown. However, the work of Carter et al. [4] suggested there may be other mechanisms of action for exogenous CHO ingestion. Cyclists performed a 40-km time trial where, in one trial, a glucose solution was infused and in the other trial a saline solution was infused [4]. Although the blood glucose concentrations and glucose disappearance were twice as high in the glucose-infused trial, there was no effect on performance. Therefore, the authors suggested there may be other reasons (beyond augmented blood glucose levels) for the ergogenic benefits of CHO ingestion.

In a subsequent study Carter et al. [5] found that mouth rinsing with a CHO solution improved performance by 2.9 % during a 1-h time trial when compared to the placebo solution. Furthermore, CHO mouth rinsing has been shown to result in higher self-selected running intensities [6] and maximal voluntary contraction [7] without any changes to circulating glucose levels. The mere presence of carbohydrates in the mouth has been postulated to influence endurance performance by ‘central factors’ as a CHO mouth rinse may involve stimulation of cortical taste neurons [5]; these receptors are thought to link with neuronal communication to pleasure centres in the brain [8]. Evidence to support this hypothesis is lacking, however, there is a suggestion that the brain can sense certain composition changes in the mouth and activation of these taste-related brain regions can influence emotion and behaviour [9]. Therefore, carbohydrate sensing in the mouth may positively impact exercise performance.

It has been reported that CHO ingestion can cause gastrointestinal (GI) discomfort and lead to decrements in endurance performance [10]. In this case mouth rinsing with a CHO solution may therefore stimulate ergogenic benefits without subsequent GI distress. However, Whitham and McKinney [11] showed that although a CHO mouth rinse did not cause GI discomfort in experienced runners, there were no performance benefits either. Other studies also report no improvements in performance from CHO mouth rinsing [12, 13]. Beelen et al. [12] examined the effects of CHO mouth rinsing 2 h after a standardised meal in well-trained cyclists. Because there were no differences in performance, power output or heart rate between placebo and CHO trials, the authors suggested that the presence of small amounts of CHO in the mouth does not impact performance when applied in more ecologically valid, postprandial conditions. However, from an evolutionary perspective, the presence of CHO in the mouth may be more pertinent when glycogen levels are compromised [12]. Nevertheless, Lane et al. [14] showed that, relative to a placebo, mouth rinsing in an unfed state showed better performance (3.4 %) than within a fed condition (1.8 %). Stannard et al. [15] found that exercising on an empty stomach enhances muscle metabolism in trained male cyclists. It is tempting to speculate that CHO mouth rinsing may therefore enhance exercise intensity (from a centrally mediating perspective) without compromising the benefits of exercising on an empty stomach; therefore allowing for an enhanced training stimulus and promoting more rapid muscular adaptations.

Numerous studies have shown the benefits of fluid ingestion on exercise performance (see Sawka et al. [16] for review). Arnaoutis et al. [17] investigated whether simply rinsing the mouth with water–the drink of choice for many athletes (many of whom simply gargle/rinse with water)–may impact performance. Their results showed that water ingestion enhanced performance in comparison to mouth rinsing, possibly via activation of the oral-pharyngeal receptors. Below et al. [18] have previously shown that fluid and CHO ingestion have independent and additive effects on endurance performance and a similar study design may therefore help to evaluate the effects of CHO mouth rinsing.

Therefore, the aim of this study was to investigate whether there are independent and/or additive effects of carbohydrate fluid mouth rinsing, placebo fluid ingestion and carbohydrate fluid ingestion on 1-h time trial cycling performance, when subjects are in a glycogen-deprived state.

## Methods

### Participants

Nine male recreationally trained cyclists and triathletes (age 32.7 ± 13.0 y, stature 1.80 ± 0.05 m, body mass (BM) 72.7 ± 7.3 kg and peak oxygen uptake ($\overset{.}{\mathrm{V}}{\mathrm{O}}_2$ peak) 55.1 ± 7.6 mL · kg^−1^ · min^−1^; mean ± SD) volunteered to participate in this study. Their training time ranged from 5 to 20 h per week, interspersed with competitive events. The study was approved by the Massey University Human Ethics Committee, Southern A (approval number 10/01). Following completion of a medical history questionnaire, written informed consent was obtained from all participants.

### Preliminary measurements

Following body mass and stature measurements, participants performed a graded exercise test, on an electronically braked cycle ergometer (model Excalibur, Lode, Groningen, Netherlands), to determine $\overset{.}{\mathrm{V}}{\mathrm{O}}_2$ peak [19]. After a 5-min warm-up at 100 W, workload was increased by 50 W every 2.5 min until a heart rate of 160 beat · min^−1^ was reached. After this point, workload was increased by 25 W every 2.5 min. Gas samples (60 s), using Douglas bag method, were collected at every 2.5 min stage. The graded exercise test continued until participants reached volitional exhaustion. The participants were asked to signal when they could go for only one more minute and a final sample was collected. The participants’ maximum power output achieved was recorded and they were verbally encouraged throughout the test. The Douglas bag samples were then analysed to determine peak $\overset{.}{\mathrm{V}}{\mathrm{O}}_2$. Participants then underwent a shortened version of the glycogen reduction exercise protocol to familiarise themselves to the relative intensities of exercise [20]. After a brief rest interval (5–10 min) participants performed a full familiarisation of the 1-h cycling time trial protocol [21]. During the performance trial the participants were introduced to the oxygen uptake measures and the mouth rinse protocol. Information on how to perform the two-day dietary record was provided after the cessation of exercise and prior to leaving the laboratory.

### Experimental trials

Participants completed four main trials, within a Latin square design, with each trial separated by seven days. Exercise was completed in an air-conditioned laboratory with no difference between trials for temperature (18.4 ± 0.2 °C to 20.2 ± 0.2 °C; *P* = 0.14) or humidity (46.4 ± 1.2 to 48.6 ± 1.4 %; *P* = 0.17). A counterbalanced design, with participants completing the trials in different orders, was used to counteract possible order effects. Participants were asked to avoid caffeine and alcohol consumption and to record dietary intake over the 48-h period prior to the first main trial and to replicate their intake prior to the other three trials. Diets were analysed for total energy intake and relative contributions of food types (FoodWorks 5.0, Xyris Software, Australia). During this time mean energy (167 ± 36 kJ · kg body mass^−1^ · day^−1^ to 196 ± 55 kJ · kg^−1^ · day^−1^; *P* = 0.57) and CHO (4.9 ± 1.2 g · kg^−1^ · day^−1^ to 5.9 ± 1.6 g · kg^−1^ · day^−1^; *P* = 0.35) intake were not different among trials. Each main trial took place over two days. On the evening of Day 1, following measurement of body mass and a fasting blood sample, participants underwent a glycogen-reducing exercise protocol followed by a low CHO meal and then a subsequent overnight fast; the following morning a performance time trial ride was conducted.

The glycogen reduction exercise was designed to reduce the glycogen content in both type I and type II muscle fibres [20]. This procedure required the participants to cycle for 30 min at 70 % peak, followed by three 50-s ‘sprints’ at double the resistive load (with 2 min rest between each bout), and then another 45 min at 70 % peak. Although muscle glycogen was not measured in the current study, this type of exercise has been shown to reduce muscle glycogen stores from 110–170 mmol glycosyl U · kg^−1^ wet wt to 13 mmol glycosyl U · kg^−1^ [22]. After completing this exercise the participants were provided with a low CHO meal which was the last meal of the day (energy content of 56 kJ · kg^−1^ BM and CHO content of 1 g · kg^−1^ BM; [23]). Thereafter, they were instructed not to consume any other food following this meal, but were allowed to ingest water ad libitum. Participants arrived in the laboratory the following morning having fasted for 10–12 h.

**Fig. 1:**
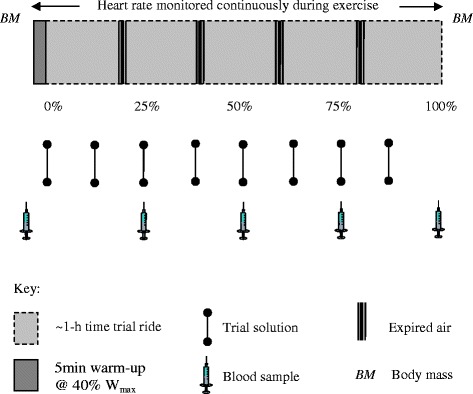
Schematic of experimental protocol. After a 5 min warm-up at 40 % of maximum power (Wmax), participants underwent the ~1-h cycling time trial. The percentage figures represent the amount of the time trial completed. The four trial solutions included **a**. 15 % carbohydrate (CHO) solution mouth rinse (0.33 mL · kg^−1^ body mass (BM)), **b** placebo (0 % CHO) mouth rinse (0.33 mL · kg^−1^ BM), **c** 7.5 % CHO solution ingestion (1.5 mL · kg^−1^ BM), and **d** placebo (0 % CHO) ingestion (1.5 mL · kg^−1^)

Figure 1 shows a schematic of the experimental protocol. Upon arrival on the morning of Day 2 participants’ nude body mass was determined and a cannula was inserted into an antecubital vein (which was kept patent by frequent flushing with sterile saline). Following a 10-ml fasting blood sample and a brief warm-up (5 min at 40 % Wmax), participants underwent the 1-h time trial [21]. Briefly, participants performed a predetermined amount of work as fast as possible based on the following formula:

$$ \mathrm{Total}\ \mathrm{work}\ \left(\mathrm{J}\right) = 0.75\kern0.5em \mathrm{Wmax}\kern0.5em 3600\ \mathrm{s} $$

The total amount of work (J) to be performed was calculated by assuming that the participants could cycle at 75 % of their maximum power output (Wmax) for 60 min [21]. Power output during the performance trial was self-selected. Participants received no verbal encouragement and no information of performance other than the amount of work completed.

### Trial solutions

Four trial solutions were used in this study. In Trial A, a 15 % CHO solution was used for rinsing and in Trial C a 7.5 % CHO solution was used for ingestion. The 15 % CHO mouth rinse solution (118 g · L^−1^ sucrose and 32 g · L^−1^ maltodextrin) and 7.5 % CHO solution (59 g · L^−1^ sucrose and 16 g · L^−1^ maltodextrin) were made up according to methods used previously [24]. The higher energy content for the mouth rinse solution was chosen as functional magnetic resonance imaging (fMRI) studies have used similar concentrations and shown changes in brain function and improvements in motor output [25]. The placebo solutions (Trials B and D) were taste and colour matched (confirmed using triangulation testing) and contained 0 % CHO and artificial sweeteners. The solutions had a mandarin flavour and were made onsite at the Massey University Albany Food Technology Laboratory and were stored in a refrigerator (Fisher and Paykell, c450, New Zealand).

During the ingestion trials 1.5 mL∙kg^−1^ body mass solutions were consumed using a sipper bottle. The participants were informed to finish the solution when it was given to them. The trial solutions were given immediately before exercise and after every 12.5 % of exercise completed (8 times in total) thus 872 ± 88 mL was consumed by participants. In the CHOI trial participants consumed 65.4 ± 6.6 g of CHO. During the mouth rinsing trials, the participants were required to rinse 0.33 mL∙kg^−1^ body mass solutions and fluids were given to the participant in a plastic volumetric syringe (Omnifix 50/60 ml Luer; Germany). Participants self-administered the mouth rinse and were asked to swirl the solution in their mouth for 8 s. After rinsing, participants expectorated all of the solution into a pre-weighed container which was then measured using electronic scales accurate to 0.0001 g (Sartorius LE3235, Germany). The expectorate volume was checked to ensure it was the same volume as what was initially provided before disposal. The mouth rinse was also administered after every 12.5 % of exercise was completed (8 times in total).

### Physiological measures

Heart rate (HR) was measured continuously during time trials using short range telemetry (Polar Electro S6101, Kempele, Finland). HR data were downloaded upon completion of the performance trial (Polar Precision Performance software version 3, Kempele, Finland). Expired air samples, using the Douglas bag technique, were taken after 20, 40, 60 and 80 % of exercise completed (1-min sample was taken after mouth piece had been inserted for 30 s to clear dead space). CO_2_ and O_2_ were determined using an analyser (Servomex 1440 Gas Analyser, Crowborough, England) prior to determination of gas volumes (Harvard Apparatus, Edenbridge, England). Using indirect calorimetry calculations, energy expenditure and fat and CHO oxidation rates during exercise were estimated.

### Blood dispensing and analyses

Ten millilitres of blood were collected prior to the glycogen reducing exercise, prior to and after every 25 % of exercise completed during the 1-h time trial. Six millilitres of the sample were collected in an EDTA tube and 4 ml were collected in a heparinised tube. To determine the haematocrit, three microhematocrit tubes were filled with heparinised blood and were then centrifuged at 10,000 rev · min^−1^ for 5 min (Haematocrit 210, Hettich, Germany). Haematocrit was measured using a microhematocrit reader (Hawkesley, Cambridge, England) and haemoglobin was determined using an automated method (Hemocue, AB, Angelholm, Sweden). The vacutainer tubes were centrifuged at 1500 g (Hanil, MF50, Korea) for 10 min at 4 °C and stored in cryotubes at −80 °C (Thermaforma 929, Nunc, Ohio, USA) for later analysis of metabolites and hormones.

Lactate was analysed via an enzymatic method using lactate oxidase (Roche Diagnostics GmbH. Switzerland; Flexor E, Vitalab Netherlands). Plasma glucose was determined using a hexokinase method (Roche Diagnostics, Basel, Switzerland; Flexor E, Vital Scientific NV, 6956 AV Spankeren/Dieren, The Netherlands). Free fatty acids (FFA) were assayed by an ACS-ACOD enzymatic method and FFA concentrations were then obtained by colorimetry (Wako Pure Chemical Industries, Ltd. Osaka, Japan and Flexor E, Vitalab Scientific NV, 6956 AV Spankeren/Dieren, The Netherlands). Insulin and C-peptide were analysed using Mercodia ultrasensitive insulin and C-peptide ELISA (a solid phase two-site enzyme immunoassay) kits, respectively. The samples were analysed in duplicate and the optical density was read at 450 nm on a plate reader (2030 Victor X, Perkin Elmer, Finland).

### Statistical analysis

The performance data were examined using mixed-model analysis of variance (ANOVA) with subjects as random effects and time and treatment as fixed effects. Data collected for most of the other variables were compared using a two-way ANOVA with repeated measures (SPSS version 18.0, Chicago, IL) to examine main effects of i) treatment (PLAR, PLAI, CHOR, CHOI) and ii) time (percentage of exercise completed) and iii) interaction of treatment x time. Mauchly’s test for sphericity was applied to the data. When sphericity was violated the Huynh-Feldt estimate was used to correct the data. One-way ANOVA was also used to examine mean data between trials (e.g. dietary composition, ambient temperature). When significant differences between the interventions were identified by ANOVA, post-hoc Student’s *t*-test, using the Holm-Bonferroni adjustment, were performed. Data are presented as means ± SD (unless otherwise indicated). Statistical significance was set at *P* < 0.05, whereas a ‘trend’ was *P* < 0.09. Practical significance was reported using effect sizes calculated from Cohen’s d. A large effect size was determined as 0.8, medium as 0.5 and small as 0.2 [26].

## Results

### Performance

**Fig. 2:**
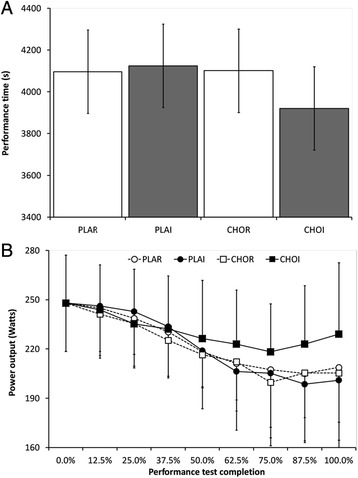
Mean (± SD) performance characteristics during 1-h cycling time trial: **a** Performance time (s) and **b** Power output (Watts). PLAR = placebo mouth rinse; PLAI = placebo ingestion; CHOR = carbohydrate mouth rinse; CHOI = carbohydrate ingestion

There was no statistical difference in performance time between trials (*P* = 0.21; Fig. 2a). However, in terms of practical significance, CHOI (3920 ± 288 s) was 4.5–5.2 % faster than other trials (4096–4124 s; Cohen’s d 0.57-0.65). Power output decreased throughout the time trial for all conditions (main effect of time, *P* < 0.01; Fig. 2b). Power output was higher in CHOI (231 ± 33 Watts) relative to other trials (221–223 Watts; main effect of treatment *P* < 0.01; Cohen’s d 0.21–0.31). There was no interaction of treatment x time (*P* = 0.41).

### Physiological and metabolic responses

**Fig. 3:**
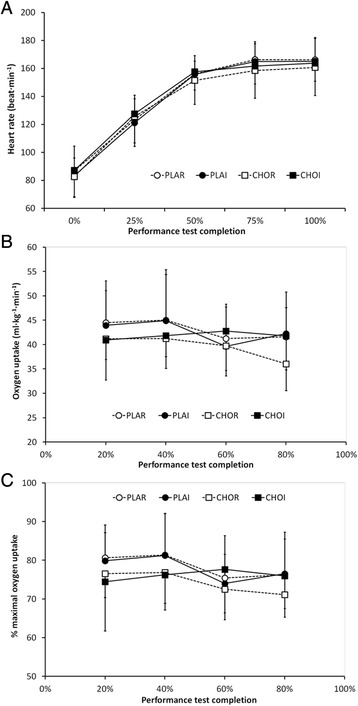
Mean (± SD) physiological responses during 1-h cycling time trial: **a** Heart rate, **b** oxygen uptake ($\overset{.}{\mathrm{V}}{\mathrm{O}}_2$) and **c** fractional utilisation of peak oxygen uptake (% $\overset{.}{\mathrm{V}}{\mathrm{O}}_2$ peak). PLAR = placebo mouth rinse; PLAI = placebo ingestion; CHOR = carbohydrate mouth rinse; CHOI = carbohydrate ingestion

There were no differences in body mass or plasma volume between the pre-glycogen reduction exercise (evening Day 1) and pre-time trial period (morning of Day 2) between trials or over time. Body mass loss was higher in the two rinsing trials (1.9 ± 0.6 % PLAR; 1.7 ± 0.4 % CHOR) relative to the ingestion trials (0.6 ± 0.3 % PLAI; 0.6 ± 0.5 % CHOI; *P* < 0.01). There were no differences between trials or over time for decrease in plasma volume as a percentage of total blood volume from pre to post-exercise. HR increased with duration of exercise (main effect of time, *P* < 0.01), but was not different between trials (*P* = 0.79; Fig. 3a). Oxygen uptake (% $\overset{.}{\mathrm{V}}{\mathrm{O}}_2$ peak) decreased throughout exercise (*P* = 0.02), but was not different between trials (*P* = 0.27; Fig. 3b). Percent $\overset{.}{\mathrm{V}}{\mathrm{O}}_2$ peak also decreased over time (*P* = 0.01), but was not different between trials (*P* = 0.60; Fig. 3c).

**Fig. 4:**
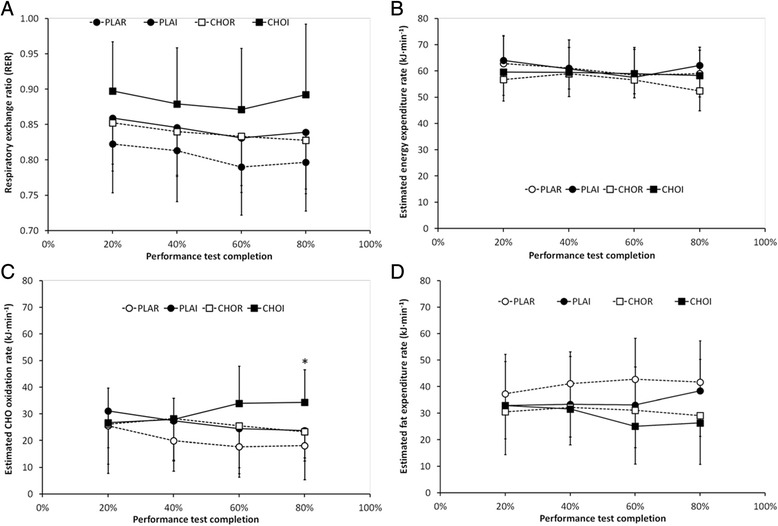
Mean (± SD) indirect calorimetry measures during 1-h cycling time trial: **a** respiratory exchange ratio (RER), **b** estimated energy expenditure rate, **c** estimated carbohydrate (CHO) oxidation rate and **d** estimated fat oxidation rate. PLAR = placebo mouth rinse; PLAI = placebo ingestion; CHOR = carbohydrate mouth rinse; CHOI = carbohydrate ingestion. * signifies higher values for CHOI relative to other trials (*P* < 0.05)

Respiratory exchange ratio (RER) was unchanged over time (*P* = 0.10) but showed a trend to be higher in CHOI (0.88 ± 0.08) relative to other trials (0.81–0.84; *P* = 0.06; Fig. 4a). Estimated energy expenditure rates showed a trend to decrease throughout exercise (*P* = 0.07), but were not different between trials (*P* = 0.10; Fig. 4b). Estimated CHO oxidation rates showed no effects of time (*P* = 0.64) or treatment (*P* = 0.14). However, there was an interaction of treatment × time (*P* = 0.049) with post-hoc analysis showing higher estimated CHO oxidation rates in CHOI relative to other trials at 80 % of completed exercise (34 ± 12 kJ · min^−1^ vs. 18–23 kJ · min^−1^; *P* < 0.05; Fig. 4c). There were no time (*P* = 0.90), treatment (*P* = 0.12) or interaction (*P* = 0.19) effects for estimated fat oxidation rates (Fig. 4d).

**Fig. 5:**
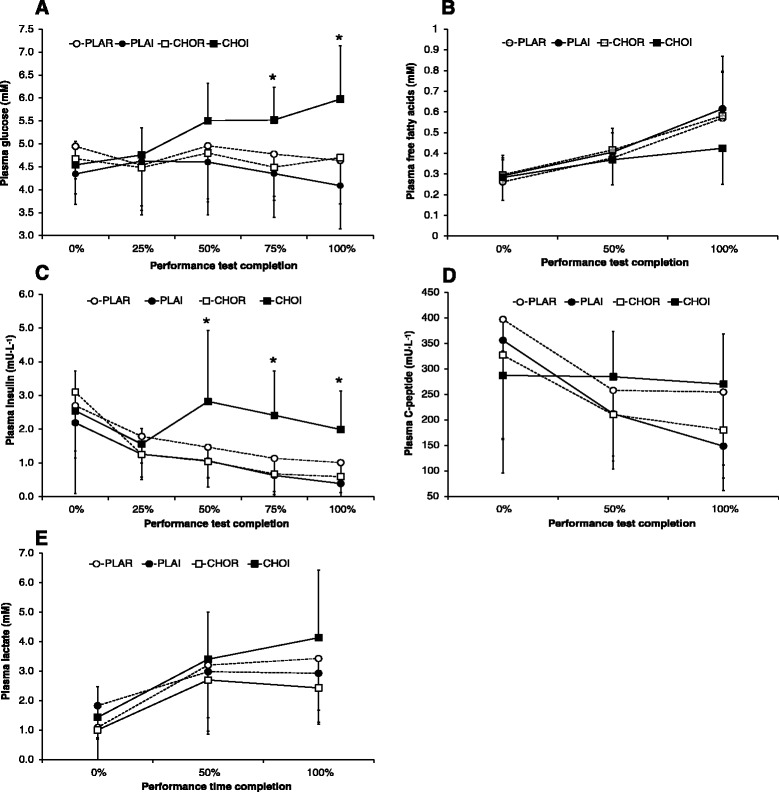
Mean (± SD) plasma metabolic markers during 1-h cycling time trial: **a** glucose, **b** free fatty acids (FFA), **c** insulin, **d** C-peptide and **e** lactate. PLAR = placebo mouth rinse; PLAI = placebo ingestion; CHOR = carbohydrate mouth rinse; CHOI = carbohydrate ingestion. * signifies higher values for CHOI relative to other trials (*P* < 0.05)

Mean plasma glucose was higher in CHOI (5.3 ± 0.9 mM) relative to other trials (4.4-4.8 mM; *P* < 0.01). Furthermore, there was an interaction of treatment x time (*P* < 0.01) with higher plasma glucose concentrations in CHOI at 75 % (5.5 ± 0.7 mM) and 100 % (6.0 ± 1.2 mM) of completed exercise relative to other trials (4.1-4.8 mM; *P* < 0.05; Fig. 5a). Plasma FFA increased over time (*P* < 0.01), with no differences between trials (*P* = 0.40) or interaction of treatment x time (*P* = 0.09; Fig. 5b). Plasma insulin concentrations decreased over time (*P* < 0.01) and were higher in the CHOI group (2.3 ± 1.4 mU · L^−1^) relative to other trials (1.1–1.6 mU · L^−1^; *P* < 0.01). Furthermore, there was an interaction of treatment x time (*P* = 0.01) with post-hoc analysis showing higher plasma insulin values in the CHOI group relative to other trials after 50, 75 and 100 % of completed exercise (*P* < 0.05; Fig. 5c). Plasma C-peptide decreased over time (*P* = 0.02), but was not different between trials (*P* = 0.10). However, C-peptide remained stable in the CHOI group, but decreased in all other trials (interaction of treatment x time, *P* = 0.02; Fig. 5d). Plasma lactate increased over time (*P* < 0.01) and was different between trials (*P* = 0.01), with post-hoc analysis showing higher values in CHOI (3.0 ± 2.0 mM) relative to CHOR (2.0 ± 1.4 mM; *P* < 0.05; Fig. 5e) only; there was no interaction of treatment x time (*P* = 0.17).

## Discussion

This study investigated the effects of CHO mouth rinse, CHO fluid ingestion, placebo mouth rinse and placebo fluid ingestion on cycling performance and metabolic and endocrine responses in subjects with low initial glycogen levels. There was no performance or metabolic stimulation with the CHO or placebo mouth rinse solutions. However, ingestion of CHO, in comparison with fluid ingestion and mouth rinse trials, increased power output during the performance test. Ingesting the CHO solution increased circulating plasma glucose, lactate and insulin concentrations, and increased estimated CHO oxidation – especially towards the latter stages of the time trial.

Mouth rinsing with CHO solutions has been promoted as a way of inducing ergogenic benefits for endurance athletes [5, 6]. However, in previous studies, under fed conditions, CHO mouth rinsing did not show any performance improvements [12], therefore indicating that the body may need to be in a glycogen-compromised state to realise any potential ergogenic benefit. The results of our study show that, even within low glycogen conditions, ingestion of a CHO solution leads to better power output and enhanced metabolic responses in comparison with simply rinsing the mouth with a CHO solution. Our results are in contrast to the findings of Lane et al. [14] who showed that CHO mouth rinsing provides performance benefits to well-trained athletes under fed or unfed conditions.

Methodological differences may help to explain some of the discrepancies between studies. Previous research that showed ergogenic benefits used CHO vs. placebo mouth rinse trials [5, 6, 14, 27] or CHO mouth rinse vs. CHO ingestion trials [28]. Our study is the first to use a comparison between fluid rinse, fluid ingestion, CHO mouth rinse and CHO ingestion trials within the same experiment (Latin square design). Participants’ cardiorespiratory fitness, as indicated by $\overset{.}{\mathrm{V}}{\mathrm{O}}_2$ max values, was higher in the aforementioned studies (61–64 mL · kg^−1^ · min^−1^) whereas in the current study the mean value was 55 mL · kg^−1^ · min^−1^. However, based on percent $\overset{.}{\mathrm{V}}{\mathrm{O}}_2$ peak, HR and lactate values, our participants were working at similar relative physiological loads. Small differences in CHO concentrations (6–7.5 %), CHO type (maltodextrin, sucrose and/or glucose) and rinsing durations (~5 s) are unlikely to explain the discrepancies between the different studies. Indeed, we incorporated a higher (15 %) CHO rinse solution and utilised an 8-s rinse protocol to maximise any potential benefit of a CHO mouth rinse. Therefore, we believe metabolic reasons may provide more plausible answers for the lack of benefit from CHO mouth rinsing.

Gant et al. [7] found that both CHO ingestion and mouth rinsing immediately facilitate corticomotor output prior to the peripheral availability of glucose. There were immediate ergogenic effects observed in maximal voluntary force and motor evoked potentials with the presence of CHO in the mouth, but no changes to circulating glucose levels. Following CHO ingestion there was peripheral appearance of blood glucose and an increase in force, however this had no effect on the primary motor cortex. It was suggested that the increase in force production observed with an increase in plasma glucose concentration after CHO ingestion was related to peripheral rather than central factors; the results of the current study certainly support this argument.

CHO ingestion has previously been shown to maintain blood glucose concentrations and CHO oxidation rates and lead to significant improvements in exercise capacity [1, 29]. In our subjects, plasma glucose levels were elevated with CHO ingestion (Fig. 5a) and power output was maintained in the CHOI group for the duration of the study (Fig. 2b). Furthermore, although estimated energy expenditure rates were unchanged between groups (Fig. 4b), the higher CHO oxidation rate (Fig. 4c) likely maintained power output in the CHOI group. As the subjects had low endogenous stores of glycogen prior to the performance time trial, it appears the exogenous CHO provision provided an impetus for higher CHO oxidation rates. Tsintzas et al. [3] suggested that when insulin and blood glucose levels were elevated muscle glycogen sparing occurred. Therefore, elevated plasma insulin (Fig. 5c) and glucose concentrations (Fig. 5a) during the CHOI trial suggests our subjects may have undergone some muscle glycogen sparing. However, without undertaking muscle biopsy, MRI and/or tracer studies assertions relating to exogenous CHO oxidation and/or glycogen sparing cannot be confirmed. Nevertheless, it appears that although CHO mouth rinsing may trigger central nervous system responses, our findings suggest that without actual ingestion of CHO, power output or metabolic changes do not take place. Therefore, contrary to previous suggestions by Beelen et al. [12], our study suggests that CHO mouth rinsing does not affect performance in an unfed state.

Insulin and C-peptide were higher in the CHOI group in comparison with the other trials (Fig. 5c and d). Insulin is the principle hormone responsible for the control of glucose metabolism and C-peptide is a strong measure of insulin secretion [30]. The C-peptide levels, reflecting insulin secretion, decreased over time during high performance exercise in all groups that did not ingest CHO. Interestingly, CHO ingestion seems to ameliorate this decline in C-peptide levels, which would contribute to the elevated insulin concentration observed in the CHOI group. The changes in insulin may help to explain the increase in power output in the CHOI group relative to the CHOR trials. It is tempting to speculate that the increase in power output could very well be driven by changes in insulin sensitivity and increased fuel utilisation. These results, together with the increase in plasma glucose levels in the CHOI group, may suggest that an increase in insulin action may drive, at least in part, the increase in performance observed in this study. The exact role of changes in insulin secretion, sensitivity or action with CHO ingestion during high-intensity exercise deserves further investigation including studies involving dynamic insulin sensitivity tests [30]. Other blood plasma parameters that may reflect changes in insulin action (e.g. leptin, glucagon-like peptide-1 or ghrelin) could also be investigated in future studies that expand inquiries into changes of metabolic regulation in this setting.

Losses of 2 % body mass during exercise have been reported to decrease endurance performance [31]. In the current study, body mass loss was greater (1.7-1.9 %) in the two mouth rinse trials compared to the two ingestion trials (0.6 %; *P* < 0.01). Dehydration has been suggested to increase glycogenolysis [32] and impact on cardiovascular function resulting in an increase in physical strain [33]. Thus, performance may have been expected to be better in the PLAI and CHOI groups relative to the mouth rinse groups. However, there was no performance, metabolic or physiological differences in the PLAI group relative to the mouth rinse groups thus suggesting that relative fluid loss and/or dehydration was not the underlying cause of fatigue in the present study. Moreover, contrary to the findings of Arnaoutis et al. [17], who used already dehydrated participants, there was no difference in performance between fluid rinse vs. fluid ingestion trials. Therefore, we speculate that the changes in power output were not simply a result of hydration status but related to the CHO ingested during high-intensity exercise.

One could argue that participants were in a fasted state which may not be realistic in a practical setting [34]. However, we believe that the present findings are an accurate representation of endurance performance as it is not uncommon for athletes who have early morning events to begin exercise in a fasted state, for example Ironman competitors [13]. Furthermore, although GI discomfort was not formally assessed in the present study, when participants were asked if they had experienced GI discomfort in the present trials there were no issues reported. Anecdotal comments suggest that many of the participants felt thirsty and dehydrated during the rinsing trials and thus preferred the ingestion trials. They reported that they were tempted to swallow the solution because of the increase in thirst that they had experienced. Subjects also found it difficult to hold their breath while they swirled the solution around in their mouth and found it hard to maintain their cadence for the 8 s during the mouth rinse process. Issues relating to social acceptability (i.e. spitting) may also deter athletes from pursuing such a method. Due to the issues of hygiene and the practical aspects, further analysis is needed to examine if sports performance mouth rinsing is possible in a ‘real life’ sporting situation. We believe that unless an athlete experiences GI distress, there seems to be no reason why one may not be able swallow the beverage.

## Conclusions

In summary, in a fasted and glycogen-reduced state, mouth rinsing with a CHO solution did not show any performance enhancements relative to a placebo control. CHO ingestion, on the other hand, enabled participants to increase power output during exercise; Cohen’s d showed moderate effect size performance differences between CHO ingestion and other trials. Plasma insulin and glucose concentrations were higher in CHO ingestion trial suggesting possible glycogen sparing. Estimated CHO oxidation rates were also higher in this trial relative to placebo control and CHO mouth rinse trials. Our data suggests that under these conditions CHO mouth rinsing was inferior to CHO ingestion for moderately trained male cyclists.
